# A genome-wide association study of breast and prostate cancer in the NHLBI's Framingham Heart Study

**DOI:** 10.1186/1471-2350-8-S1-S6

**Published:** 2007-09-19

**Authors:** Joanne M Murabito, Carol L Rosenberg, Daniel Finger, Bernard E Kreger, Daniel Levy, Greta Lee Splansky, Karen Antman, Shih-Jen Hwang

**Affiliations:** 1 The National Heart, Lung, and Blood Institute's Framingham Heart Study, Framingham, MA, USA; 2 Section of General Internal Medicine and the Sections of Hematology/Oncology, Department of Medicine, Boston University School of Medicine, Boston, MA, USA; 3 National Heart, Lung, and Blood Institute, Bethesda, MD, USA

## Abstract

**Background:**

Breast and prostate cancer are two commonly diagnosed cancers in the United States. Prior work suggests that cancer causing genes and cancer susceptibility genes can be identified.

**Methods:**

We conducted a genome-wide association study (Affymetrix 100K SNP GeneChip) of cancer in the community-based Framingham Heart Study. We report on 2 cancer traits – prostate cancer and breast cancer – in up to 1335 participants from 330 families (54% women, mean entry age 33 years). Multivariable-adjusted residuals, computed using Cox proportional hazards models, were tested for association with qualifying SNPs (70, 987 autosomal SNPs with genotypic call rate ≥80%, minor allele frequency ≥10%, Hardy-Weinberg test p ≥ 0.001) using generalized estimating equations (GEE) models and family based association tests (FBAT).

**Results:**

There were 58 women with breast cancer and 59 men with prostate cancer. No SNP associations attained genome-wide significance. The top SNP associations in GEE models for each trait were as follows: breast cancer, rs2075555, p = 8.0 × 10^-8 ^in *COL1A1*; and prostate cancer, rs9311171, p = 1.75 × 10^-6 ^in *CTDSPL*. In analysis of selected candidate cancer susceptibility genes, two *MSR1 *SNPs (rs9325782, GEE p = 0.008 and rs2410373, FBAT p = 0.021) were associated with prostate cancer and three *ERBB4 *SNPs (rs905883 GEE p = 0.0002, rs7564590 GEE p = 0.003, rs7558615 GEE p = 0.0078) were associated with breast cancer. The previously reported risk SNP for prostate cancer, rs1447295, was not included on the 100K chip. Results of cancer phenotype-genotype associations for all autosomal SNPs are web posted at http://www.ncbi.nlm.nih.gov/projects/gap/cgi-bin/study.cgi?id=phs000007.

**Conclusion:**

Although no association attained genome-wide significance, several interesting associations emerged for breast and prostate cancer. These findings can serve as a resource for replication in other populations to identify novel biologic pathways contributing to cancer susceptibility.

## Background

Breast and prostate cancer are the most frequently diagnosed cancers in women and men respectively with over 200,000 cases each of new breast and prostate cancer estimated for 2006 in the United States [1]. Furthermore, prostate cancer is the second leading cause of cancer-related deaths in men and breast cancer is the second leading cause of cancer-related deaths in women. Family history is a well established risk factor for both breast and prostate cancer providing evidence for underlying genetic factors contributing to cancer occurrence. Accumulating research has identified a number of candidate genes and biologic pathways associated with increased susceptibility to cancer. However, even the most penetrant mutations, such as in *BRCA1 *and *BRCA2*, account for only 5–10% of cases and are present in <1% of the general population. Genome-wide association studies (GWAS) provide a comprehensive approach to identification of genetic variants associated with cancer risk unconstrained by existing knowledge and may permit detection of common genetic variants each with small associated cancer risk but great public health impact. Reports from two recent GWAS demonstrated the importance of this approach with the discovery of novel loci for breast cancer susceptibility [2,3]. Four SNPs in the *FGFR2 *gene were strongly associated with breast cancer and the association was confirmed in a sample of cases and controls derived from three additional studies [3].

We used the Framingham Heart Study (FHS) Affymetrix 100K SNP genotyping resource for GWAS of breast and prostate cancer phenotypes. The FHS offers the advantage of a prospective longitudinal family-based community sample with participants who have been well-characterized throughout adulthood with respect to risk factors and diseases, including cancer. We report results of two complementary strategies to identify genome-wide associations with cancer phenotypes: 1) a simple low p-value SNP ranking strategy; and 2) 100K SNP associations within candidate genes and regions previously reported to be associated with these cancers in humans.

## Methods

### Study sample

The genotyped study sample comprised 1345 Original cohort (n = 258) and Offspring (n = 1087) participants from the 330 largest FHS families. The Overview [4] provides further details of this sample. There were 250 participants in the sample with cancer (excluding non-melanoma skin cancer) including 58 women with breast cancer, and 59 men with prostate cancer. The Boston University Medical Center Institutional Review Board approved the examination content of Original Cohort and Offspring examinations. All participants provided written informed consent including consent for genetic studies.

### Cancer phenotype definitions and residual creation

The 5209 Original Cohort participants have been examined biennially since study inception in 1948 and the 5124 Offspring Cohort participants (children of the Original Cohort and spouses of the children) have been examined approximately every 4 years since enrollment in 1971. Cancer cases were identified at routine examinations or by health-history updates for participants who did not attend an examination. Medical records were reviewed by two independent reviewers (BEK, GLS). The vast majority of cancers were confirmed by pathology reports; <3.4% of cancer cases were based on death certificate or clinical diagnosis alone. The 1976 World Health Organization ICD-O coding was used to classify all primary cancers. Hence, topography, location (subdivision of site), histology or morphology (cell histopathology), behavior (degree of malignancy), and grade (histological grading & differentiation) were recorded along with date of diagnosis. Cancer cases reviewed through December 31, 2005 were included in this study. The proportion of women and men in the study sample with breast (8%) and prostate cancer (9%) respectively was similar to that in the full FHS sample.

Cox proportional hazards models were used to generate martingale residuals using the PHREG procedure in SAS to perform the regression analysis of time from study entry to cancer diagnosis or last contact free of cancer. Breast cancer was examined in women only and models were cohort-specific and adjusted for 1) age at entry and 2) age, parity, and body mass index at study entry. For prostate cancer, in men only, models were cohort-specific and adjusted for age at entry.

### Genotyping

Affymetrix 100K SNP GeneChip genotyping and the Marshfield STR genotyping performed by the Mammalian Genotyping Service http://research.marshfieldclinic.org/genetics are described in the Overview [4]. SNPs were excluded if minor allele frequency <0.10 (n = 38062); genotypic call rate <0.80 (n = 2346); Hardy Weinberg equilibrium test p < 0.001 (n = 1595). There were 70,987 autosomal SNPs available for analysis after the exclusions.

### Statistical Analysis

The statistical methods for genome-wide association analyses are described in detail in the Overview [4]. While there are various suggested methods for interpretation of genome-wide significance, we chose to use a conservative (p < 0.05/10^-6 ^= 5 × 10^-8^) threshold to define genome-wide significance for this report.

### Association

All cancer residual traits listed in Table 1 were computed using Cox proportional hazards models. The full set of FHS participants with the phenotype were used to create the residuals. The residuals were used to test for association between the genotyped subset of participants and the SNPs using family-based association test (FBAT) and generalized estimating equation (GEE) models. FBAT analyses were restricted to at least 10 informative families. The GEE tests tended to give an excess of very small p-values over what would be expected (see Overview [4]).

**Table 1:** Cancer Phenotypes for the Framingham Heart Study 100K Analyses

Phenotype*	Traits	Number of participants	Events	Adjustment (database phenotype variable name)
All Cancer* (excluding non-melanoma skin cancer)	2	1335	250	Cohort- and sex-specific 1. age at study entry (allcancer1) 2. multivariable adjusted for age, body mass index, and cigarette smoking at study entry (allcancer2)
Breast Cancer (women)	2	723	58	Cohort-specific 1. age at study entry (breastcancer1) 2. multivariable adjusted for age, parity, and body mass index at study entry (breastcancer2)
Prostate Cancer (men)	1	617	59	Cohort-specific 1. age at study entry (prostatecancer1)

Residuals from these models were used as traits to test for association with SNP genotypes. All residuals created using Cox Proportional Hazards regression. Cancers ascertained through December 31, 2005

*All SNP associations for all phenotypes in the table including all cancer are available on the web http://www.ncbi.nlm.nih.gov/projects/gap/cgi-bin/study.cgi?id=phs000007

### SNP prioritization

We used several strategies to prioritize SNPs associated with cancer traits. First, we used an untargeted approach whereby SNP associations were ranked according to the strength of the p-value for each trait. Next we identified candidate genes reported to be associated with each cancer trait from review of the literature. Candidate genes were selected by searching PubMed (using susceptibility, gene, cancer and (breast or prostate) as keywords, last accessed 08-15-06), and the Entrez Gene and Online Mendelian Inheritance in Man resources, as well as recent text books. All available 100K SNPs in or near the a priori selected candidate genes were investigated for association with cancer traits. Finally for prostate cancer, we also examined SNP associations in the region on chromosome 8 (8q24) previously reported to be associated with prostate cancer in Icelandic families and confirmed in three case-control series [5] and African American men [6]. Further, for prostate cancer we examined the overlap in SNP associations in our study and the top 500 ranked SNPs from the Cancer Genetics Markers of Susceptibility (CGEMS) project sponsored by the National Cancer Institute. Because CGEMS used an Illumina platform for genotyping and the genotyping used in this study was performed with an Affymetrix platform, the gene_symbol from the UCSC annotation was used to link with CGEMS top 500 SNP list. Using this method, 1487 SNPs in FHS 100K (including SNPs with MAF < 0.1) correlated with the CGEMS top 500 SNPs related to a known gene.

SNPs were annotated with the UCSC genome browser tables using the May 2004 assembly http://genome.ucsc.edu/[7,8]. All genes within 60 kb of the top ranked SNPs were identified. The physical location of the SNPs was based on Build 35 of Genome for this report; however, the 100K web browser was based on Build 36.

## Results

The cancer phenotypes available in the FHS 100K SNP resource, including details of the sample size, number of cancer events, and covariate adjustment for each trait are listed in Table 1. In this report, we consider only two phenotypes: breast cancer in women (multivariable-adjusted) and prostate cancer in men. Among participants in the 100K sample the mean age at breast cancer diagnosis was 59 years (range 35 to 83 years) in Offspring Cohort women and 70 years (range 35 to 97 years) in Original Cohort women; the mean age at prostate cancer diagnosis was 66 years (range 43 to 85 years) in Offspring Cohort men and 76 years (range 53 to 95 years) in Original Cohort men.

For each of the cancer phenotypes, Table 2 provides the top 15 SNPs ranked in order by lowest p-value for the GEE models and for the FBAT models (all SNP associations can be viewed on the web) [9]. None of the SNP associations achieved genome-wide significance (p < 5 × 10^-8^) [4]. However, for prostate cancer, the top SNP in GEE models, rs9311171, is in *CTDSPL *(CTD {carboxy-terminal domain, RNA polymerase II, polypeptide A} small phosphatase-like), a gene that may play a role in tumor suppression [10].

**Table 2:** Cancer Phenotypes for FHS 100K Project: Results of Association Analyses*

2a. GEE results ranked by p-value					
**SNP**	**Chr**	**Physical Position**	**GEE p-value**	**FBAT p-value**	**Gene region (within 60 kb)**

**BREAST CANCER**					

rs2075555	17	45,629,290	**8.3 × 10**^-8^	0.157	*COL1A1*
rs6556756	5	163,821,858	**5.0 × 10**^-7^	0.114	
rs1154865	12	72,276,104	**6.6 × 10**^-7^	0.052	
rs1978503	18	51,815,280	**9.66 × 10**^-7^	0.287	
rs1926657	13	94,672,957	**1.9 × 10**^-6^	0.072	*ABCC4*
rs10263639	7	66,503,417	**2.7 × 10**^-6^	0.590	
rs10490113	2	59,410,998	**4.7 × 10**^-6^	0.077	
rs458685	21	30,099,382	**6.0 × 10**^-6^	0.064	*GRIK1*
rs1876206	15	46,687,878	**6.0 × 10**^-6^	0.742	*FBN1*
rs9314033	5	163,822,784	**1.2 × 10**^-5^	0.058	
rs10486490	7	25,846,789	**1.3 × 10**^-5^	0.245	
rs9325024	5	146,185,979	**1.7 × 10**^-5^	0.112	*PPP2R2B*
rs1294255	1	229,808,093	**1.9 × 10**^-5^	0.470	
rs10513754	3	178,681,862	**2.8 × 10**^-5^	0.189	
rs10501093	11	27,946,695	**3.0 × 10**^-5^	0.066	*KIF18A*

**PROSTATE CANCER**					

rs9311171	3	37,971,481	**1.8 × 10**^-6^	0.392	*CTDSPL*
rs1529276	13	102,726,008	**1.8 × 10**^-6^	0.071	
rs10498792	6	51,774,590	**3.0 × 10**^-6^	0.285	*PKHD1*
rs4466137	5	83,021,495	**3.1 × 10**^-6^	0.305	*HAPLN1*
rs345013	3	146,656,486	**5.1 × 10**^-6^	0.002	
rs10505137	8	111,210,785	**1.3 × 10**^-5^	0.002	
rs1873038	3	175,020,343	**1.8 × 10**^-5^	0.317	*NLGN1*
rs8019932	14	29,676,177	**1.9 × 10**^-5^	0.019	
rs344985	3	146,619,475	**2.1 × 10**^-5^	0.002	
rs1920676	11	15,789,323	**2.2 × 10**^-5^	0.196	
rs10511206	10	127,209,365	**2.4 × 10**^-5^	0.028	
rs1037569	5	119,954,204	**2.8 × 10**^-5^	0.165	
rs2109312	2	128,292,061	**3.0 × 10**^-5^	0.786	*WDR33*
rs1352416	3	174,963,409	**3.4 × 10**^-5^	0.201	*NLGN1*
rs1440714	11	15,,790,399	**3.7 × 10**^-5^	0.223	

**2b. FBAT results ranked by p-value**					

**SNP**	**Chr**	**Physical Position**	**GEE p-value**	**FBAT p-value**	**Gene Region (within 60 kb)**

**BREAST CANCER**					

rs7711990	5	180,307,504	0.010	**8.4 × 10**^-5^	
rs1451125	4	137,449,995	0.008	**9.5 × 10**^-5^	
rs4266352	4	109,823,951	0.001	**1.2 × 10**^-4^	
rs6720918	2	122,873,276	0.027	**1.9 × 10**^-4^	
rs6101183	20	59,025,355	0.132	**2.0 × 10**^-4^	
rs9307064	4	90,503,827	0.159	**2.2 × 10**^-4^	
rs10512287	9	101,538,523	0.807	**2.3 × 10**^-4^	*GRIN3A*
rs7190881	16	7,368,424	0.475	**2.9 × 10**^-4^	
rs2224402	1	229,359,076	0.418	**3.1 × 10**^-4^	*C1orf57*
rs10516690	4	84,822,792	0.954	**3.7 × 10**^-4^	
rs10487920	7	145,895,727	0.085	**3.9 × 10^-4^**	*CNTNAP2*
rs6479347	9	91,348,121	0.017	**4.5 × 10^-4^**	
rs1924587	13	96,195,380	0.020	**4.6 × 10^-4^**	*HS6ST3*
rs2059273	16	5,991,144	0.005	**4.9 × 10^-4^**	*A2BP1*
rs2822669	21	14,752,449	0.029	**5.7 × 10^-4^**	*SAMSN1*

**PROSTATE CANCER**					

rs657057	6	56,402,241	0.109	**3.4 × 10**^-5^	*DST*
rs6691482	1	170,274,521	0.007	**4.0 × 10**^-5^	*SLC9A11*
rs4378061	9	10,930,127	0.633	**6.6 × 10**^-5^	
rs2056387	1	234,250,153	0.159	**1.1 × 10**^-4^	*RYR2*
rs2274626	1	176,253,589	0.005	**1.4 × 10**^-4^	*NPHS2*
rs181247	17	53,562,730	0.552	**3.2 × 10**^-4^	*OR4D1|OR4D2*
rs7827348	8	3,020,222	0.054	**3.6 × 10**^-4^	
rs255561	5	82,386,566	0.040	**3.9 × 10**^-4^	*XRCC4*
rs1504858	4	137,063,555	0.010	**3.9 × 10**^-4^	
rs954709	4	167,317,424	0.049	**4.2 × 10**^-4^	
rs2179443	20	37,450,516	0.057	**4.4 × 10**^-4^	
rs35239	5	68,064,834	0.657	**4.6 × 10**^-4^	
rs477516	8	16,758,362	0.057	**5.1 × 10**^-4^	
rs595725	1	28,993,331	0.239	**5.3 × 10**^-4^	*OPRD1*
rs10515322	5	101,539,543	0.051	**5.4 × 10**^-4^	*SLCO4C1*

*Autosomal SNPs with genotypic call rate ≥80%, minor allele frequency ≥10%, Hardy-Weinberg test p ≥ 0.001, and ≥10 informative families for FBAT. The physical location of the SNPs was based on Build 35 of Genome; however, the 100K web browser was based on Build 36

There were several additional associations not listed in Table 2 that were of interest. For prostate cancer, in GEE models rs906304 (rank 27, p = 0.000067), is in *NCOR2 *also known as *SMRT*. SMRT levels have been reported to be elevated in prostate cancer cells, and result in suppression of anti-proliferative target gene actions for the vitamin D receptor [11,12]. In FBAT models, for prostate cancer SNP rs255561 (rank 17, p = 0.00039), is near *XRCC4*, a gene that plays a role in DNA repair and rs1897676 (rank 50, p = 0.0012), is in *PTPRD*. Protein tyrosine phosphatases are signaling molecules involved in the regulation of a variety of cellular processes including cell growth, differentiation, mitotic cycle, and oncogenic transformation [13]. For breast cancer in GEE models rs4146372 (rank 31, p = 0.00007), is near *RAB21*, SNP rs9307561 (rank 40, p = 0.0001), is near *FAT4*, and rs10512849 (rank 46, p = 0.00014), is in *FGF10*. These genes appear to play biologic roles in a variety of processes including tumor growth and suppression [13,14]. In FBAT models, for breast cancer rs2836391 (rank 46, p = 0.0012), is in *ERG*, an oncogene important in the development of prostate cancer [15,16].

**Table 3:** All SNP Associations within Selected Breast and Prostate Candidate Genes (up to 60 kb)

Associations within Candidate Genes Selected by Cancer Type: FBAT or GEE p-value < 0.01						
**Cancer Trait**	**Gene**	**SNP**	**Chr**	**Physical Position**	**GEE p-value**	**FBAT p-value**

Breast cancer	*ERBB4*	rs905883	2	213,013,976	2.0 × 10^-4^	0.8517
Breast cancer	*ERBB4*	rs7564590	2	213,213,406	0.0032	0.2567
Breast cancer	*ERBB4*	rs7558615	2	212,771,655	0.008	0.8457
Prostate cancer	*MSR1*	rs9325782	8	16,134,844	8.2 × 10^-4^	0.3448
Prostate cancer	*MSR1*	rs2410373	8	15,968,877	0.014	0.02051

Our second strategy was to identify from the literature candidate genes implicated in breast and prostate cancer susceptibility (see Additional data file 1). For prostate cancer, we identified 63 candidate genes. Twenty of these candidate genes had from 1 to 20 SNPs on the 100K chip whereas the remaining genes had no SNP coverage on the chip. For breast cancer, 75 potential candidate genes were identified, 28 of these genes had between 1 and 35 SNPs on the 100K chip and the remaining candidate genes were not covered on the chip. Two SNPs in *MSR1 *(rs9325782, GEE p = 0.008 and rs2410373, FBAT p = 0.021) were associated with prostate cancer and three SNPs in *ERBB4 *(rs905883 GEE p = 0.0002, rs7564590 GEE p = 0.003, rs7558615 GEE p = 0.0078) were associated with breast cancer (Table 3). For prostate cancer, a region on chromosome 8q24 was recently reported to be associated with prostate cancer risk in Icelandic men and confirmed in three case-control series of men of European ancestry and African American men [5,6]. There were a total of 64 SNPs on the 100K chip in this 8q24 region (128 to 129.3 Mb interval). However, the reported risk SNP, rs1447295, was not included on the 100K chip and none of the 64 available SNPs were in linkage disequilibrium with the risk SNP. Five other SNPs in this region were associated with prostate cancer with a GEE or FBAT p-value < 0.01 (Table 4).

**Table 4:** SNPs in the Chromosome 8q24 region Associated with Prostate Cancer:GEE or FBAT p-value < 0.01

SNP	Physical Position	Distance from reported marker	MAF	GEE p-value	FBAT p-value
rs7001069	128179828	-253268	0.0188	3.0 × 10^-5^	0.39322
rs10505483	128194377	-238719	0.0223	4.0 × 10^-5^	0.32697
rs1562871	128470954	37858	0.1696	0.16618	0.00328
rs10505474	128486686	53590	0.406	0.00995	0.0121
rs10505506	129114473	681377	0.3275	0.00829	0.92941

The National Cancer Institute commenced the CGEMS [17] initiative to conduct genome-wide association studies to identify genetic factors related to prostate and breast cancer. We examined overlap between the top 500 ranked SNPs for prostate cancer in CGEMS phase 1a [18] and the results of the FHS 100K GWAS analysis for prostate cancer. The physical position of the SNP was used to detect overlapping associations and the results are shown in Table 5. Of note, many of the associations in Table 5 are in SNPs with very low minor allele frequencies and the results are presented according to minor allele frequency. *WWOX *gene, a tumor suppressor gene, that has been reported to play a role in prostate cancer [19], showed evidence of association (rs3751832, p = 0.0009) in our study sample.

**Table 5:** Prostate Cancer SNP Associations Common to Both CGEMS Top 500 Ranked SNPs and FHS 100K SNPs

CGEMS Phase 1a					FHS 100K					
**DBSNP_ID**	**Chr**	**Physical Position**	**CGEMS p-value**	**CGEMS Rank**	**100K SNP**	**Physical Position**	**GEE p-value**	**FBAT p-value**	**MAF**	**Nearest Gene**

**Minor Allele Frequency ≥10%**										

rs3017183	18	51,319,241	0.00024100	85	rs3794889	51,212,935	0.017	0.799	0.224	*TCF4*
rs3017183	18	51,319,241	0.00024100	85	rs4801149	51,214,227	0.044	0.983	0.221	*TCF4*
rs2253319	21	35,109,916	0.00041100	137	rs2834645	35,109,556	0.009	0.293	0.206	*RUNX1*
rs392715	16	24,023,323	0.00041800	141	rs10492797	24,122,453	0.036	0.407	0.175	*PRKCB1*
rs10256504	7	3,516,796	0.00092200	295	rs4418248	3,194,181	1.8 × 10^-4^	0.056	0.109	*SDK1*
rs6102912	20	40,636,349	0.00111000	350	rs986831	40,594,892	0.009	0.094	0.407	*PTPRT*
rs4782742	16	81,573,151	0.00127400	395	rs254315	82,313,913	7.1 × 10^-4^	0.011	0.141	*CDH13*
rs2371438	2	212,791,037	0.00128500	401	rs10497958	212,771,465	0.055	0.047	0.164	*ERBB4*
rs6577648	8	135,555,688	0.00144900	445	rs10505624	135,607,862	4.9 × 10^-4^	0.007	0.147	*ZFAT1*
rs9327886	5	102,931,646	0.00154600	468	rs10515347	102,950,833	0.006	0.062	0.354	*NUDT12*
rs6555491	5	7,799,792	0.00158700	478	rs10512920	7,455,561	0.009	0.647	0.244	*ADCY2*

**Minor Allele Frequency <10%**										

rs38276	7	14,078,240	0.00055700	181	rs10486031	14,258,703	1.7 × 10^-5^	0.759	0.032	*DGKB*
rs216666	2	80,745,015	0.00057000	184	rs10520247	79,693,452	2.8 × 10^-5^	0.240	0.065	*CTNNA2*
rs216666	2	80,745,015	0.00057000	184	rs10520246	79,693,324	1.5 × 10^-4^	0.198	0.050	*CTNNA2*
rs1261256	5	22,490,853	0.00060200	193	rs10520880	22,634,003	8.5 × 10^-5^	0.356	0.065	*CDH12*
rs7610584	3	171,635,885	0.00061900	196	rs10513681	171,624,010	7.8 × 10^-7^	0.402	0.025	*CLDN11*
rs7329659	13	97,872,564	0.00064700	209	rs7989050	97,680,299	6.0 × 10^-6^	0.258	0.014	*FARP1*
rs208354	7	2,554,104	0.00065000	211	rs10487577	2,583,719	2.0 × 10^-7^	N/A	0.012	*GNA12*
rs3751832	16	77,771,773	0.00079900	257	rs10514443	77,353,386	9.3 × 10^-4^	N/A	0.008	*WWOX*

CGEMS = Cancer Genetics Markers of Susceptibility; MAF = minor allele frequency; N/A= not available, less than 10 informative families for FBAT

## Discussion

Breast and prostate cancer are the two most frequently diagnosed cancers in the United States and result in substantial morbidity and mortality [1]. A number of breast and prostate cancer susceptibility genes and chromosomal regions have been identified [2,3,5,20-34]. However, currently known genes account for only a fraction of the familial aggregation of breast cancer [25] and few prostate cancer susceptibility genes have even been identified. Risk for these cancers is likely mediated through variation in many genes, each conferring a relatively small risk for the disease. Genome-wide association studies provide an opportunity to discover novel genes and pathways that play a causal role in cancer occurrence and in turn may lead to new therapies for the prevention and treatment of cancer. Finding genetic associations with breast and prostate cancer risk that are robust across multiple studies may facilitate the identification of high risk individuals who can be targeted for early screening and preventive interventions.

We report GWAS results for breast cancer and prostate cancer phenotypes in a community-based sample of adults from two generations of the same families. Although none of the SNP associations achieved genome-wide significance in GEE or FBAT models, this resource has the potential to detect novel cancer susceptibility genes and to explore the relevance of promising candidate gene associations to human cancer. Our results can be compared to those from other genome-wide association studies such as the National Cancer Institute's CGEMS [17]. Although the two studies used different genotyping platforms limiting overlap in the SNPs examined, we were able to determine the physical position of the SNPs. Using this strategy, SNPs in the *ERRB4 *gene (CGEMS DSSNP_ID rs2371438 and FHS 100K SNP rs10497958) were associated with prostate cancer. ErbB proteins are widely expressed in prostate cells [35] and may play a role in tumor development, growth and progression in human prostate cancer [36,37]. We also examined the 8q24 region previously associated with prostate cancer risk. CGEMS investigators recently reported a second independent risk SNP (rs6983267) within the 8q24 strongly associated with prostate cancer [34]. The Affymetrix 100K GeneChip did not include either of the previously reported risk SNPs; however, we did identify five other SNPs in this region associated with prostate cancer. The underlying biologic mechanism mediating prostate cancer risk associated with the SNPs and chromosomal region remains unknown. A two-stage approach, genome-wide association followed by selective genotyping of SNPs with suggestive evidence of association, may provide an efficient strategy for pursuing initial genome-wide results [2,38,39].

Several important limitations merit comment. First, this study used cancer cases identified through surveillance of a multigenerational community-based sample. The enrollment and examination of Original Cohort and Offspring Cohort participants began years before DNA collection occurred. Thus, a survival bias may have been introduced. Our cases may be comprised of early-staged and less lethal cancers. To address this potential bias, we adjusted for covariates using the full Framingham sample, and used the residual traits for the subset of individuals genotyped using the 100K Affymetrix GeneChip to test for association with the SNPs in linear regression models. Residual traits from Cox models typically are not ideally distributed for linear regression models, but our adjustment method using the full Framingham sample precludes the testing of SNP associations with cancer traits using Cox models. Second, we had a small number of cancer events (250 all cancer cases, 58 breast cancer cases and 59 prostate cancer cases) limiting our ability to detect SNP associations. In a recent small GWAS of age-related macular degeneration that included 96 cases and 50 controls, an association with the *CFH *gene was identified [40] and confirmed in larger studies [41-43]. However, in that report, individuals homozygous for the *CFH *risk allele had a sevenfold increased likelihood of age-related macular degeneration [40]. It is very unlikely that common genetic variants for cancer phenotypes will confer a risk for cancer susceptibility of that magnitude. For example, the odds ratio associated with the risk marker identified for prostate cancer in region 8q24 was 1.72 in the combined Icelandic sample [5]. Furthermore, the associations between prostate cancer and the SNPs with low minor allele frequency (Table 5) are likely to be false positive associations given the small number of prostate cancer cases in our sample. Third, the 100K Affymetrix GeneChip provides limited coverage of the genome; many of our a priori candidate genes did not have any SNP coverage on the chip and coverage of some candidate genes that were present on the chip was suboptimal. Importantly, the replicated risk SNP, rs1447295, for prostate cancer [5] was not included on the chip. NHLBI has committed funds for a 550 K genome-wide scan on all FHS participants. This will enable us to confirm our initial 100K SNP associations in a larger sample with a greater number of cancer cases and with denser coverage of the genome. We did not examine epistasis or gene-environment interactions which may modify the associations noted in this study. Lastly, most of our associations are likely to be due to chance. Replication studies are needed to determine if any of the results we report are indicative of true associations. It is important that our data be used in conjunction with data from other samples given the high probability of false positive associations.

## Conclusion

In summary, the untargeted genome-wide approach to detect genetic associations for cancer traits provides an opportunity to identify novel biologic pathways related to cancer occurrence and to direct future study of candidate genes that hold the most promise for relevance to cancer risk in humans. Enhancing our understanding of the mechanisms responsible for cancer susceptibility may in turn identify novel strategies for early detection, prevention, and treatment of breast and prostate cancers. These data serve as a resource for replication in other population-based samples.

## Abbreviations

CGEMS = cancer genetics markers of susceptibility; FBAT = family-based association test; FHS = Framingham Heart Study; GEE = generalized estimating equations; GWAS = genome-wide association study; ICD-O = international classification of diseases for oncology; MAF = minor allele frequency; SNP = single nucleotide polymorphism.

## Competing interests

The authors declare that they have no competing interests.

## Authors' contributions

All authors have made substantial contributions to conception and design or acquisition of phenotypic data. JMM, SJW, DF, DL, CLR contributed to the analysis and interpretation of data. JMM, CLR, DL, DF, and SJW have been involved in drafting the manuscript or revising it critically for important intellectual content and all authors have read and approved the final manuscript.

## Supplementary Material

Additional file 1 List of selected candidate genes for breast and prostate cancer. Click here for file

## References

[B1] American Cancer Society Cancer Facts and Figures 2006 2006 Atlanta: American Cancer Society

[B2] Easton DF Pooley KA Dunning AM Pharoah PD Thompson D Ballinger DG Struewing JP Morrison J Field H Luben R Wareham N Ahmed S Healey CS Bowman R Luccarini C Conroy D Shah M Munday H Jordan C Perkins B West J Redman K Meyer KB Haiman CA Kolonel LK Henderson BE Le Marchand L Brennan P Sangrajrang S Gaborieau V Odefrey F Shen CY Wu PE Wang HC Eccles D Evans DG Peto J Fletcher O Johnson N Seal S Stratton MR Rahman N Chenevix-Trench G Bojesen SE Nordestgaard BG Axelsson CK Garcia-Closas M Brinton L Chanock S Lissowska J Peplonska B Nevanlinna H Fagerholm R Eerola H Kang D Yoo KY Noh DY Ahn SH Hunter DJ Hankinson SE Cox DG Hall P Wedren S Liu J Low YL Bogdanova N Schurmann P Dork T Tollenaar RA Jacobi CE Devilee P Klijn JG Sigurdson AJ Doody MM Alexander BH Zhang J Cox A Brock IW Macpherson G Reed MW Couch FJ Goode EL Olson JE Meijers-Heijboer H van den OA Uitterlinden A Rivadeneira F Milne RL Ribas G Gonzalez-Neira A Benitez J Hopper JL McCredie M Southey M Giles GG Schroen C Justenhoven C Brauch H Hamann U Ko YD Spurdle AB Beesley J Chen X Aghmesheh M Amor D Andrews L Antill Y Armes J Armitage S Arnold L Balleine R Begley G Beilby J Bennett I Bennett B Berry G Blackburn A Brennan M Brown M Buckley M Burke J Butow P Byron K Callen D Campbell I Chenevix-Trench G Clarke C Colley A Cotton D Cui J Culling B Cummings M Dawson SJ Dixon J Dobrovic A Dudding T Edkins T Eisenbruch M Farshid G Fawcett S Field M Firgaira F Fleming J Forbes J Friedlander M Gaff C Gardner M Gattas M George P Giles G Gill G Goldblatt J Greening S Grist S Haan E Harris M Hart S Hayward N Hopper J Humphrey E Jenkins M Jones A Kefford R Kirk J Kollias J Kovalenko S Lakhani S Leary J Lim J Lindeman G Lipton L Lobb L Maclurcan M Mann G Marsh D McCredie M McKay M Anne MS Meiser B Milne R Mitchell G Newman B O'loughlin I Osborne R Peters L Phillips K Price M Reeve J Reeve T Richards R Rinehart G Robinson B Rudzki B Salisbury E Sambrook J Saunders C Scott C Scott E Scott R Seshadri R Shelling A Southey M Spurdle A Suthers G Taylor D Tennant C Thorne H Townshend S Tucker K Tyler J Venter D Visvader J Walpole I Ward R Waring P Warner B Warren G Watson E Williams R Wilson J Winship I Young MA Bowtell D Green A Defazio A Chenevix-Trench G Gertig D Webb P Mannermaa A Kosma VM Kataja V Hartikainen J Day NE Cox DR Ponder BA Genome-wide association study identifies novel breast cancer susceptibility loci Nature 2007 in press 10.1038/nature05887 PMC2714974 17529967

[B3] Hunter DJ Kraft P Jacobs KB Cox DG Yeager M Hankinson SE Wacholder S Wang Z Welch R Hutchinson A Wang J Yu K Chatterjee N Orr N Willett WC Colditz GA Ziegler RG Berg CD Buys SS McCarty CA Feigelson HS Calle EE Thun MJ Hayes RB Tucker M Gerhard DS Fraumeni JF Jr Hoover RN Thomas G Chanock SJ A genome-wide association study identifies alleles in FGFR2 associated with risk of sporadic postmenopausal breast cancer Nat Genet 2007 in press 10.1038/ng2075 PMC3493132 17529973

[B4] Cupples LA Arruda H Benjamin EJ D'Agostino RB Sr Demissie S DeStefano AL Dupuis J Falls K Fox CS Gottlieb D Govindaraju DR Guo CY Heard-Costa N Hwang SJ Katherisan S Kiel D Laramie JM Larson MG Levy D Liu CY Lunetta KL Mailman M Manning AK Meigs JB Murabito JM Newton-Cheh C O'Connor GT O'Donnell CJ Pandey MA Seshadri S Vasan RS Wang ZY Wilk JB Wolf PA Yang Q Atwood LD The Framingham Heart Study 100K SNP genome-wide association study resource: Overview of 17 phenotype working group reports BMC Med Genet 2007 8 Suppl 1 S1 17903291 10.1186/1471-2350-8-S1-S1 PMC1995613

[B5] Amundadottir LT Sulem P Gudmundsson J Helgason A Baker A Agnarsson BA Sigurdsson A Benediktsdottir KR Cazier JB Sainz J Jakobsdottir M Kostic J Magnusdottir DN Ghosh S Agnarsson K Birgisdottir B Le Roux L Olafsdottir A Blondal T Andresdottir M Gretarsdottir OS Bergthorsson JT Gudbjartsson D Gylfason A Thorleifsson G Manolescu A Kristjansson K Geirsson G Isaksson H Douglas J Johansson JE Balter K Wiklund F Montie JE Yu X Suarez BK Ober C Cooney KA Gronberg H Catalona WJ Einarsson GV Barkardottir RB Gulcher JR Kong A Thorsteinsdottir U Stefansson K A common variant associated with prostate cancer in European and African populations Nat Genet 2006 38 652 658 10.1038/ng1808 16682969

[B6] Freedman ML Haiman CA Patterson N McDonald GJ Tandon A Waliszewska A Penney K Steen RG Ardlie K John EM Oakley-Girvan I Whittemore AS Cooney KA Ingles SA Altshuler D Henderson BE Reich D Admixture mapping identifies 8q24 as a prostate cancer risk locus in African-American men Proc Natl Acad Sci USA 2006 103 14068 14073 10.1073/pnas.0605832103 16945910 PMC1599913

[B7] Karolchik D Baertsch R Diekhans M Furey TS Hinrichs A Lu YT Roskin KM Schwartz M Sugnet CW Thomas DJ Weber RJ Haussler D Kent WJ The UCSC Genome Browser Database Nucleic Acids Res 2003 31 51 54 10.1093/nar/gkg129 12519945 PMC165576

[B8] Kent WJ Sugnet CW Furey TS Roskin KM Pringle TH Zahler AM Haussler D The human genome browser at UCSC Genome Res 2002 12 996 1006 Article published online before print in May 2002 10.1101/gr.229102 12045153 PMC186604

[B9] 2007 http://www.ncbi.nlm.nih.gov/projects/gap/cgi-bin/study.cgi?id=phs000007

[B10] Kashuba VI Li J Wang F Senchenko VN Protopopov A Malyukova A Kutsenko AS Kadyrova E Zabarovska VI Muravenko OV Zelenin AV Kisselev LL Kuzmin I Minna JD Winberg G Ernberg I Braga E Lerman MI Klein G Zabarovsky ER RBSP3 (HYA22) is a tumor suppressor gene implicated in major epithelial malignancies Proc Natl Acad Sci USA 2004 101 4906 4911 10.1073/pnas.0401238101 15051889 PMC387347

[B11] Khanim FL Gommersall LM Wood VH Smith KL Montalvo L O'Neill LP Xu Y Peehl DM Stewart PM Turner BM Campbell MJ Altered SMRT levels disrupt vitamin D3 receptor signalling in prostate cancer cells Oncogene 2004 23 6712 6725 10.1038/sj.onc.1207772 15300237

[B12] Abedin SA Banwell CM Colston KW Carlberg C Campbell MJ Epigenetic corruption of VDR signalling in malignancy Anticancer Res 2006 26 2557 2566 16886664

[B13] 2006 http://www.ncbi.nlm.nih.gov/entrez/query.fcgi?db=gene&cmd

[B14] Theodorou V Boer M Weigelt B Jonkers J van d V Hilkens J Fgf10 is an oncogene activated by MMTV insertional mutagenesis in mouse mammary tumors and overexpressed in a subset of human breast carcinomas Oncogene 2004 23 6047 6055 10.1038/sj.onc.1207816 15208658

[B15] Tomlins SA Rhodes DR Perner S Dhanasekaran SM Mehra R Sun XW Varambally S Cao X Tchinda J Kuefer R Lee C Montie JE Shah RB Pienta KJ Rubin MA Chinnaiyan AM Recurrent fusion of TMPRSS2 and ETS transcription factor genes in prostate cancer Science 2005 310 644 648 10.1126/science.1117679 16254181

[B16] Liu W Chang B Sauvageot J Dimitrov L Gielzak M Li T Yan G Sun J Sun J Adams TS Turner AR Kim JW Meyers DA Zheng SL Isaacs WB Xu J Comprehensive assessment of DNA copy number alterations in human prostate cancers using Affymetrix 100K SNP mapping array Genes Chromosomes Cancer 2006 45 1018 1032 10.1002/gcc.20369 16897747

[B17] 2006 http://cgems.cancer.gov/about/executive_summary.asp

[B18] 2006 https://caintegrator.nci.nih.gov/cgems/

[B19] Qin HR Iliopoulos D Semba S Fabbri M Druck T Volinia S Croce CM Morrison CD Klein RD Huebner K A role for the WWOX gene in prostate cancer Cancer Res 2006 66 6477 6481 10.1158/0008-5472.CAN-06-0956 16818616

[B20] Xu J Dimitrov L Chang BL Adams TS Turner AR Meyers DA Eeles RA Easton DF Foulkes WD Simard J Giles GG Hopper JL Mahle L Moller P Bishop T Evans C Edwards S Meitz J Bullock S Hope Q Hsieh CL Halpern J Balise RN Oakley-Girvan I Whittemore AS Ewing CM Gielzak M Isaacs SD Walsh PC Wiley KE Isaacs WB Thibodeau SN McDonnell SK Cunningham JM Zarfas KE Hebbring S Schaid DJ Friedrichsen DM Deutsch K Kolb S Badzioch M Jarvik GP Janer M Hood L Ostrander EA Stanford JL Lange EM Beebe-Dimmer JL Mohai CE Cooney KA Ikonen T Baffoe-Bonnie A Fredriksson H Matikainen MP Tammela TL Bailey-Wilson J Schleutker J Maier C Herkommer K Hoegel JJ Vogel W Paiss T Wiklund F Emanuelsson M Stenman E Jonsson BA Gronberg H Camp NJ Farnham J Cannon-Albright LA Seminara D A combined genomewide linkage scan of 1,233 families for prostate cancer-susceptibility genes conducted by the international consortium for prostate cancer genetics Am J Hum Genet 2005 77 219 229 10.1086/432377 15988677 PMC1224525

[B21] Kammerer S Roth RB Reneland R Marnellos G Hoyal CR Markward NJ Ebner F Kiechle M Schwarz-Boeger U Griffiths LR Ulbrich C Chrobok K Forster G Praetorius GM Meyer P Rehbock J Cantor CR Nelson MR Braun A Large-scale association study identifies ICAM gene region as breast and prostate cancer susceptibility locus Cancer Res 2004 64 8906 8910 10.1158/0008-5472.CAN-04-1788 15604251

[B22] Xu J Zheng SL Hawkins GA Faith DA Kelly B Isaacs SD Wiley KE Chang B Ewing CM Bujnovszky P Carpten JD Bleecker ER Walsh PC Trent JM Meyers DA Isaacs WB Linkage and association studies of prostate cancer susceptibility: evidence for linkage at 8p22–23 Am J Hum Genet 2001 69 341 350 10.1086/321967 11443539 PMC1235306

[B23] Kraft P Pharoah P Chanock SJ Albanes D Kolonel LN Hayes RB Altshuler D Andriole G Berg C Boeing H Burtt NP Bueno-de-Mesquita B Calle EE Cann H Canzian F Chen YC Crawford DE Dunning AM Feigelson HS Freedman ML Gaziano JM Giovannucci E Gonzalez CA Haiman CA Hallmans G Henderson BE Hirschhorn JN Hunter DJ Kaaks R Key T Le Marchand L Ma J Overvad K Palli D Pike MC Riboli E Rodriguez C Setiawan WV Stampfer MJ Stram DO Thomas G Thun MJ Travis R Trichopoulou A Virtamo J Wacholder S Genetic variation in the HSD17B1 gene and risk of prostate cancer PLoS Genet 2005 1 e68 10.1371/journal.pgen.0010068 16311626 PMC1287955

[B24] Breast Cancer Association Consortium Commonly studied single-nucleotide polymorphisms and breast cancer: results from the Breast Cancer Association Consortium J Natl Cancer Inst 2006 98 1382 1396 17018785 10.1093/jnci/djj374

[B25] Antoniou AC Easton DF Models of genetic susceptibility to breast cancer Oncogene 2006 25 5898 5905 10.1038/sj.onc.1209879 16998504

[B26] Smith P McGuffog L Easton DF Mann GJ Pupo GM Newman B Chenevix-Trench G Szabo C Southey M Renard H Odefrey F Lynch H Stoppa-Lyonnet D Couch F Hopper JL Giles GG McCredie MR Buys S Andrulis I Senie R Goldgar DE Oldenburg R Kroeze-Jansema K Kraan J Meijers-Heijboer H Klijn JG van Asperen C van L I Vasen HF Cornelisse CJ Devilee P Baskcomb L Seal S Barfoot R Mangion J Hall A Edkins S Rapley E Wooster R Chang-Claude J Eccles D Evans DG Futreal PA Nathanson KL Weber BL Rahman N Stratton MR A genome wide linkage search for breast cancer susceptibility genes Genes Chromosomes Cancer 2006 45 646 655 10.1002/gcc.20354 16575876 PMC2714969

[B27] Walsh T Casadei S Coats KH Swisher E Stray SM Higgins J Roach KC Mandell J Lee MK Ciernikova S Foretova L Soucek P King MC Spectrum of mutations in BRCA1, BRCA2, CHEK2, and TP53 in families at high risk of breast cancer JAMA 2006 295 1379 1388 10.1001/jama.295.12.1379 16551709

[B28] Berndt SI Chatterjee N Huang WY Chanock SJ Welch R Crawford ED Hayes RB Variant in sex hormone-binding globulin gene and the risk of prostate cancer Cancer Epidemiol Biomarkers Prev 2007 16 165 168 10.1158/1055-9965.EPI-06-0689 17220347

[B29] Bergman A Karlsson P Berggren J Martinsson T Bjorck K Nilsson S Wahlstrom J Wallgren A Nordling M Genome-wide linkage scan for breast cancer susceptibility loci in Swedish hereditary non-BRCA1/2 families: suggestive linkage to 10q23.32–q25.3 Genes Chromosomes Cancer 2007 46 302 309 10.1002/gcc.20405 17171685

[B30] Rahman N Seal S Thompson D Kelly P Renwick A Elliott A Reid S Spanova K Barfoot R Chagtai T Jayatilake H McGuffog L Hanks S Evans DG Eccles D Easton DF Stratton MR PALB2, which encodes a BRCA2-interacting protein, is a breast cancer susceptibility gene Nat Genet 2007 39 165 167 10.1038/ng1959 17200668 PMC2871593

[B31] Cox A Dunning AM Garcia-Closas M Balasubramanian S Reed MW Pooley KA Scollen S Baynes C Ponder BA Chanock S Lissowska J Brinton L Peplonska B Southey MC Hopper JL McCredie MR Giles GG Fletcher O Johnson N Dos SSI Gibson L Bojesen SE Nordestgaard BG Axelsson CK Torres D Hamann U Justenhoven C Brauch H Chang-Claude J Kropp S Risch A Wang-Gohrke S Schurmann P Bogdanova N Dork T Fagerholm R Aaltonen K Blomqvist C Nevanlinna H Seal S Renwick A Stratton MR Rahman N Sangrajrang S Hughes D Odefrey F Brennan P Spurdle AB Chenevix-Trench G Beesley J Mannermaa A Hartikainen J Kataja V Kosma VM Couch FJ Olson JE Goode EL Broeks A Schmidt MK Hogervorst FB Veer LJ Kang D Yoo KY Noh DY Ahn SH Wedren S Hall P Low YL Liu J Milne RL Ribas G Gonzalez-Neira A Benitez J Sigurdson AJ Stredrick DL Alexander BH Struewing JP Pharoah PD Easton DF A common coding variant in CASP8 is associated with breast cancer risk Nat Genet 2007 39 352 358 10.1038/ng1981 17293864

[B32] Camp NJ Farnham JM Cannon-Albright LA Localization of a prostate cancer predisposition gene to an 880-kb region on chromosome 22q12.3 in Utah high-risk pedigrees Cancer Res 2006 66 10205 10212 10.1158/0008-5472.CAN-06-1233 17047086

[B33] Lange EM Ho LA Beebe-Dimmer JL Wang Y Gillanders EM Trent JM Lange LA Wood DP Cooney KA Genome-wide linkage scan for prostate cancer susceptibility genes in men with aggressive disease: significant evidence for linkage at chromosome 15q12 Hum Genet 2006 119 400 407 10.1007/s00439-006-0149-6 16508751

[B34] Yeager M Orr N Hayes RB Jacobs KB Kraft P Wacholder S Minichiello MJ Fearnhead P Yu K Chatterjee N Wang Z Welch R Staats BJ Calle EE Feigelson HS Thun MJ Rodriguez C Albanes D Virtamo J Weinstein S Schumacher FR Giovannucci E Willett WC Cancel-Tassin G Cussenot O Valeri A Andriole GL Gelmann EP Tucker M Gerhard DS Fraumeni JF Jr Hoover R Hunter DJ Chanock SJ Thomas G Genome-wide association study of prostate cancer identifies a second risk locus at 8q24 Nat Genet 2007 39 645 649 10.1038/ng2022 17401363

[B35] Nasu K Tanji N Nishioka R Wang J Yanagihara Y Ozawa A Yokoyama M Sakayama K EXpression of ErbB proteins in human prostate Arch Androl 2006 52 185 190 10.1080/01485010500316238 16574600

[B36] Soung YH Lee JW Kim SY Wang YP Jo KH Moon SW Park WS Nam SW Lee JY Yoo NJ Lee SH Somatic mutations of the ERBB4 kinase domain in human cancers Int J Cancer 2006 118 1426 1429 10.1002/ijc.21507 16187281

[B37] Gallo RM Bryant I Fry R Williams EE Riese DJ Phosphorylation of ErbB4 on Tyr1056 is critical for inhibition of colony formation by prostate tumor cell lines Biochem Biophys Res Commun 2006 349 372 382 10.1016/j.bbrc.2006.08.055 16934755 PMC1618953

[B38] Skol AD Scott LJ Abecasis GR Boehnke M Joint analysis is more efficient than replication-based analysis for two-stage genome-wide association studies Nat Genet 2006 38 209 213 10.1038/ng1706 16415888

[B39] Wang H Thomas DC Pe'er I Stram DO Optimal two-stage genotyping designs for genome-wide association scans Genet Epidemiol 2006 30 356 368 10.1002/gepi.20150 16607626

[B40] Klein RJ Zeiss C Chew EY Tsai JY Sackler RS Haynes C Henning AK SanGiovanni JP Mane SM Mayne ST Bracken MB Ferris FL Ott J Barnss C Hoh J Complement factor H polymorphism in age-related macular degeneration Science 2005 308 385 389 10.1126/science.1109557 15761122 PMC1512523

[B41] Despriet DD Klaver CC Witteman JC Bergen AA Kardys I de Maat MP Boekhoorn SS Vingerling JR Hofman A Oostra BA Uitterlinden AG Stijnen T van Duijn CM de Jong PT Complement factor H polymorphism, complement activators, and risk of age-related macular degeneration JAMA 2006 296 301 309 10.1001/jama.296.3.301 16849663

[B42] Edwards AO Ritter R III Abel KJ Manning A Panhuysen C Farrer LA Complement factor H polymorphism and age-related macular degeneration Science 2005 308 421 424 10.1126/science.1110189 15761121

[B43] Schaumberg DA Christen WG Kozlowski P Miller DT Ridker PM Zee RY A prospective assessment of the Y402H variant in complement factor H, genetic variants in C-reactive protein, and risk of age-related macular degeneration Invest Ophthalmol Vis Sci 2006 47 2336 2340 10.1167/iovs.05-1456 16723442 PMC1828123

