# Supplementary material for: A genome-wide association study of breast and prostate cancer in the NHLBI's Framingham Heart Study
Source: BMC Med Genet. 2007 Sep 19;8(Suppl 1):S6. doi: 10.1186/1471-2350-8-S1-S6 (PMC1995609; doi:10.1186/1471-2350-8-S1-S6)
Supplement: Additional file 1 — List of selected candidate genes for breast and prostate cancer. [file 1471-2350-8-S1-S6-S1.doc]

**List of Selected Candidate Genes for Breast and Prostate Cancer**

| **Selected Candidate Genes for Prostate Cancer*** | | |
| --- | --- | --- |
| **Gene Name** | **Gene** | **# SNPs on 100K chip** |
| AT motif-binding factor 1 | *ATBF1* | 3 |
| B-Cell CLL/lymphoma 2 | *BLC2* | 3 |
| Breast cancer 2, early onset | *BRCA2* | 2 |
| Cadherin 1 | *CDH1* | 2 |
| Checkpoint Kinase 2 | *CHEK2* | 3 |
| Cytochrome p450, family 19, subfamily A, polypeptide 1 | *CYP19A1* | 20 |
| ElaC, E. Coli, homolog 2 | *ELAC2* | 1 |
| Eph tyrosine kinase 3 (ephrin receptor eph B2) | *EPHB2* | 2 |
| Glucoaminyl (N-acetyl) transferase 1, core 2 | *GCNT1* | 2 |
| Hypoxia-inducible factor 1 alpha subunit | *HIF1A* | 2 |
| Huntington Interacting Protein 1 | *HIP1* | 4 |
| Hydroxysteroid (17-beta) dehydrogenase 3 | *HSD17B3* | 1 |
| Mitotic arrest deficienct 1, yeast homologue-like 1 | *MAD1L1* | 3 |
| Macrophage scavenger receptor - 1 | *MSR1* | 8 |
| MAX-interacting protein-1 | *MXI1* | 10 |
| Poly (ADP ribose) polymerase family, member 1 | *PARP1* | 1 |
| Phosphatase and tensin homolog | *PTEN* | 1 |
| Serpin peptidase inhibitor, clade B, member 10 | *SERPINB10* | 2 |
| Serpin peptidase inhibitor, clade B, member 2 | *SERPINB2* | 4 |
| Vitamin D Receptor | *VDR* | 4 |

*An additional 43 selected genes for prostate cancer had 0 SNPs on the 100K chip

| **Selected Candidate Genes for Breast Cancer*** | | |
| --- | --- | --- |
| **Gene Name** | **Gene** | **# SNPs on 100K chip** |
| Ataxia-telangiectasia mutated | *ATM* | 2 |
| BRCA1 associated ring domain 1 | *BARD1* | 3 |
| B-Cell CLL/lymphoma 2 | *BCL2* | 3 |
| Breast cancer 2 ,early onset | *BRCA2* | 2 |
| BRCA1 interacting protein C terminal helicase 1 | *BRIP1* | 3 |
| Catalase | *CAT* | 2 |
| Cyclin-dependent kinase inhibitor 2A | *CDKN2A* | `1 |
| CHK2 Checkpoint Homolog | *CHEK2* | 3 |
| cytochrome p450, family 19, subfamily A, polypeptide 1 | *CYP19A1* | 20 |
| Epidermal growth factor | *EGF* | 2 |
| v-erb-a Erythroblastic Leukemia Viral oncogene homolog 4 | *ERBB4* | 35 |
| Excision repair cross complementing rodent repair deficiency, comp. Group 5 | *ERCC5* | 1 |
| Estrogen receptor 1 | *ESR1* | 9 |
| Estrogen receptor 2 (ER beta) | *ESR2* | 1 |
| Low density lipoprotein-related rotein 1 | *LRP1* | 1 |
| Matrix metallopeptidase 1 | *MMP1* | 3 |
| Nuclear receptor co-activator 3 | *NCOA3* | 2 |
| Nuclear mitotic apparatus protein 1 | *NUMA1* | 3 |
| Progesterone receptor | *PGR* | 3 |
| Phosphatidylinositol 3-kinase, catalytic, alpha polypeptide | *PIK3CA* | 1 |
| Presenilin 2 | *PSEN2* | 4 |
| Phosphatase and tensin homolog | *PTEN* | 1 |
| Protein tyrosine phosphatase, receptor type, J | *PTPRJ* | 1 |
| RAD51 homolog (Rec. A homolog, E. Coli) | *RAD51* | 2 |
| Transforming growth factor alpha | *TGFA* | 10 |
| Thioredoxin | *TXN* | 2 |
| Thioredoxin reductase 2 | *TXNRD2* | 4 |
| Vitamin D receptor | *VDR* | 2 |

*An additional 47 genes selected for breast cancer had 0 SNPs on the 100k chip.
